# Association between the Type of Diabetes Treatment and Depressive Symptoms among Patients with Diabetes: A Cross-Sectional Study of Korea Community Health Surveys Data, 2011–2016

**DOI:** 10.3390/ijerph16224441

**Published:** 2019-11-12

**Authors:** Hyeon Ji Lee, Jieun Jang, Sang Ah Lee, Sarah Soyeon Oh, Eun-Cheol Park

**Affiliations:** 1 Department of Public Health, Graduate School, Yonsei University, Seoul 03722, Korea; 2 Institute of Health Services Research, Yonsei University, Seoul 03722, Korea; 3 Research and Analysis Team, National Health Insurance Service Ilsan Hospital, Goyang 10444, Korea; 4 Department of Preventive Medicine, College of Medicine, Yonsei University, Seoul 03722, Korea

**Keywords:** diabetes mellitus, depression, therapeutics, drug therapy, insulin

## Abstract

The purpose of this study was to examine the association between the different types of treatment for diabetes and depressive symptoms. In particular, this study assessed the presence of depressive symptoms in patients with diabetes who are undergoing pharmacological treatments in terms of sex. This study used data from the 2011–2016 Korea Community Health Survey, which included responses from 50,774 male and 48,978 female participants with diabetes who were receiving pharmacological treatments. Patients aged ≥30 years were included. Logistic regression analysis was conducted to examine the significance of the association. Male participants treated with insulin injection were more likely to experience depressive symptoms than those taking oral hypoglycemic (oral agents) only (odds ratio (OR) = 1.27; 95% confidence interval (CI): 1.04–1.56). Male patients treated with both oral agents and insulin injection had the highest OR value of depressive symptoms among different types of treatments (OR = 1.41, 95% CI: 1.25–1.60). The same tendency was observed in female participants. In female patients, however, the association between depressive symptoms and insulin injection was statistically insignificant (both oral agents and insulin injection OR = 1.35, 95% CI: 1.22–1.50, insulin injection OR = 1.17, 95% CI: 0.98–1.41). The association between depressive symptoms and the type of diabetes treatment was more significant in male than in female patients. Those who were treated with oral agents and insulin injection were more likely to have depressive symptoms than those receiving oral agents of treatment.

## 1. Introduction

Diabetes is a non-communicable disease [1]. However, its prevalence is increasing worldwide [2]. According to International Diabetes Federation (IDF) data, the number of individuals with diabetes worldwide in 2017 was approximately 424.9 million, with a prevalence rate of 8.8%. Moreover, in 2045, its prevalence is predicted to increase up to 9.9% among 6.37 billion adults worldwide [3]. The increase in its prevalence is a cause of concern because diabetes is one of the major causes of death. According to the World Health Organization (WHO) data, diabetes is the sixth leading cause of death in 2015, with 1.59 million recorded cases [4]. The leading cause of death among diabetes patients is diabetes complications. 

Blood glucose levels that are poorly managed increase the risk of these complications [5]. In other words, uncontrolled diabetes can cause complications, which can threaten the lives of patients. Therefore, the blood glucose levels (pharmacological or non-pharmacological treatment) of patients with diabetes must be controlled. If the blood glucose levels of patients with diabetes is properly controlled, diabetes-related complications and death can be prevented [3]. 

In contrast, depression increases the risk of complications and death among individuals with diabetes. Depression is a well-known comorbidity in patients with chronic diseases, and diabetes is not an exception [6]. Distress from the condition, depressive mood or symptoms, and clinical depression can be observed in patients with diabetes [7]. Depressive symptoms in patients with diabetes reduces adherence to treatments [8] and disrupts the management of high blood glucose. Moreover, they can cause complications and increase the risk of mortality from diabetes [3,9]. Diabetes patients with depression have approximately 1.5 times increased risk of mortality compared to diabetes patients without depression [10]. Thus, depression is fatal in these patients. 

In several previous studies, diabetes and depressive symptoms had a bidirectional association [11]. Both are risk factors that cause each other to occur, and they also exacerbate each other’s disease state. Therefore, individuals with diabetes have twice the increased risk for depression than those without [12,13]. In particular, among patients with diabetes, emotional distress (including depressive symptoms) is more commonly observed in women than in men [14,15]. Approximately 80% of patients with diabetes are more likely to experience a recurrence of depressive symptoms [16]. Furthermore, treatments for high blood glucose had a negative impact on depression [17,18,19,20]. 

According to previous studies, the degree of depressive symptoms among patients with diabetes differed according to the type of treatment for diabetes. Studies that used the problem areas in diabetes (PAID) questionnaire focused on depression, and the emotional problems of the patients with diabetes showed different emotional distresses, which depends on the type of treatment for type 2 diabetes [15]. In another study that used the PAID questionnaire, the scores of the IDDM (insulin dependent diabetes mellitus) and NIDDM (non-insulin-dependent diabetes mellitus) groups differed in terms of the type of diabetes treatment [21]. In addition, in a study by Black on older Mexican Americans with diabetes, the current type of diabetes treatment was associated with depressive symptoms [22]. The study by Hermanns showed the difference in the proportion of individuals with type 1 and 2 diabetes without insulin treatment and those with type 2 diabetes with insulin treatment who were diagnosed with clinical and subclinical depression [23].

Therefore, the present study aimed to examine the association between the different types of diabetes treatment and depressive symptoms. In particular, the depressive symptoms of patients with diabetes who were on pharmacologic treatments (oral agents, insulin injection, and both oral agent medications and insulin injection) were assessed in terms of sex. 

## 2. Materials and Methods 

### 2.1. Data Collection and Participants

This study used data from The Korea Community Health Survey (CHS) in 2011–2016. The CHS is a cross-sectional, annual, nationwide public health survey, which has been performed by the Korea Center for Disease Control and Prevention (KCDC) since 2008. The surveyed population is registered adult residents aged 19 years and older in the community health center. The sample design is a complex, stratified, multistage, probability-cluster sampling design. The survey is conducted by a trained interviewer in a one-on-one interview in Korean through computer-assisted personal interview (CAPI) [24]. The CHS data are openly published. Additionally, participants’ information was fully anonymized and unidentified prior to analysis. Accordingly, ethical approval was not required for this study. Ethical approval of the CHS was obtained from the KCDC (2010-02CON-22-P; 2011-05CON-04-C; 2012-07CON-01-2C; 2013-06EXP-01-3C; 2014-08EXP-09-4C-A; 2016-10-01-P-A). The dataset is available on The CHS website (https://chs.cdc.go.kr/chs/index.do).

The total number of respondents in this survey was 1,372,650. However, only respondents aged 30 years or older (*n* = 1,220,729) were selected. Two-stage filtering was performed to identify patients with diabetes who were undergoing treatment. First, patients who answered yes to the question “Have you ever been diagnosed with diabetes by a doctor?” were included (*n* = 128,474). Second, only those who answered yes to the question “Are you currently being treated?” were included among them (*n* = 115,990). There were no missing data in the questionnaire that asked for the types of diabetes treatment. Individuals with missing data on covariate variables were further excluded. Therefore, a final sample population of 99,752 was included in the analysis. Of these, 50,774 were male and 48,978 were female. 

### 2.2. Dependent Variable

Self-reported depressive symptom was considered as a dependent variable. The following question was included in the questionnaire: “Have you ever felt sad or desperate enough to cause disruption to your daily life over two consecutive weeks in the last year?” The respondents were classified who answered yes as having depressive symptoms and those who answered no as having no depressive symptoms. Those who responded with “Don’t know” and “No response” were excluded.

### 2.3. Independent Variable

The type of diabetes treatment used to control blood glucose was considered an independent variable. This study used the CHS question “What is the treatment you are doing to manage your blood glucose? Please answer all” to set the interest variable. The possible responses to the questions include the following: insulin injections (yes, no), oral agent medications (oral agents; yes, no), non-pharmacologic therapy (exercise, diet; yes, no). Among the patients, those who answered that they were only treated with non-pharmacological therapy were excluded to examine the association between the different type of pharmacological diabetes treatments and depression.

### 2.4. Control Variables

The covariates were as follows: age, socioeconomic (SES) factors, health-related factors, and the year of the survey. For SES factors, educational level, marital status, occupation (white collar, pink collar, blue collar, and others), monthly household income, and region of residence were considered. The monthly household income was categorized into six groups. At the start of the investigation, 1000 KRW was equivalent to 0.93 USD [25]. Health-related factors included alcohol status (whether they drink more than one cup of alcohol during their lifetime), smoking status (whether they smoke more than five packs during their lifetime), walking activities, perceived health status, perceived stress, body mass index, and a history or diagnosis of hypertension and dyslipidemia. Walking activities were assessed by using the following questionnaire: “How many days did you walk at least 10 minutes at a time in the last week?” The responses were classified as follows: None or 1 day per week as inactive, 2–3 days a week as low, 4–5 days a week as middle, and 6–7 days a week as high.

### 2.5. Statistical Analysis

χ^2^ test and logistics regression analysis were used for the analysis. All the analyses were performed by classifying the patients according to sex (male and female). χ^2^ test was used to examine the significant difference in depressive symptoms depending on the different types of pharmacological treatments for diabetes and to control covariates, such as age, SES factors, health-related factors, and year. A *p* value <0.05 was considered statistically significant. Logistic regression analysis was conducted to determine adjusted odds ratios (ORs) and 95% confidence intervals (CIs). Subgroup analysis was performed according to the different types of treatments for diabetes and depressive symptoms. Statistical analyses were performed by using the Statistical Analysis System software version 9.4 (SAS Institute Inc., Cary, NC, USA). 

## 3. Results

### 3.1. Study Population

Table 1 shows the general characteristics of the study population. Of the total diabetes patients who were undergoing diabetes treatment, 90,182 (90.4%) were treated with oral agents and 7049 (7.1%) were treated with oral agents and insulin injection. Insulin injection was the least used (2521; 2.5%). Among the male and female diabetes patients, 2911 (2.9%) and 5207 people (5.2%) had depressive symptoms, respectively, indicating a higher proportion of women affected. As for the depressive symptoms according to the type of treatment, in men, treatment with both oral agents and insulin injection were the most frequently used at 5.7%, followed by insulin injection at 5.1%. Oral agents were the least frequently used at 2.6%. In women, treatment with both oral agents and insulin injection were the most frequently used at 8.2%, followed by insulin injection at 6.2%. Oral agents were the least frequently used at 5.0%.

### 3.2. Association between Type of Diabetes Treatment and Depressive Symptoms

Table 2 shows the results of the factors associated with the depressive symptoms of the diabetes patients. The logistic regression analyses adjusted for covariates revealed that the type of diabetes treatment was related to the depressive symptoms of the diabetes patients. In addition, the type of diabetes treatment was more closely related to the symptoms of depression in men with diabetes than in women with diabetes. Compared with taking oral agents, in both men and women, insulin injections more likely caused depressive symptoms. However, the results of women were not significant as compared with those of men (OR = 1.27; 95% CI: 1.04–1.56 vs OR = 1.17, 95% CI: 0.98–1.41). The male and female diabetes patients who received oral agents and insulin injections were the most likely to have depressive symptoms than the diabetes patients with one type of treatment. This showed statistically significant results for both men and women (OR = 1.41, 95% CI: 1.25–1.60 and OR = 1.35, 95% CI: 1.22–1.50, respectively).

### 3.3. Results of Subgroup Analyses

Table 3 shows the results of the subgroup analyses of the association of age, educational level, walking activity, and body mass index with depressive symptoms. In most cases, in both men and women, insulin injection showed higher OR values than the reference value for oral agents, but many results of the subgroup analyses were not significant. In both men and women, oral agents and insulin injection together had higher OR values than the reference value for oral agents, with most of the patients showing significant results. The male patients treated with oral agents and insulin injection showed that the age of 40 to 49 had the highest OR values (OR = 1.89, 95% CI: 1.28–2.81) and the age of 60–69 had the lowest OR values (OR = 1.28, 95% CI: 1.02–1.62). In body mass index, the OR value of underweight was not significant and that of normal weight was the lowest (OR = 1.36, 95% CI: 1.11–1.67). The female treated with oral agents and insulin injection showed a similar trend to the male except for body mass index. The age of 40–49 had the highest OR value (OR = 1.54, 95% CI: 1.04–2.28), and the results for the age of 60–69 were not significant. In body mass index, the OR value of underweight was not significant. Normal weight had the highest OR value (OR = 1.46, 95% CI: 1.22–1.74), overweight had a lower OR value (OR = 1.35, 95% CI: 1.08–1.67), and obesity had the lowest OR value (OR = 1.27, 95% CI: 1.07–1.50). This was a tendency different from the results of men.

## 4. Discussion

This study investigated the association between the types of treatment and depressive symptoms among diabetes patients undergoing diabetes treatment by dividing sex, using nationally represented data. The depressive symptoms in both male and female patients treated with diabetes varied according to the type of diabetes treatment. In all diabetes patients undergoing diabetes treatment, women reported more depressive symptoms than men. However, in examining the factors of depressive symptoms, it was found that men were more affected by depressive symptoms than women, depending on the type of diabetes treatment. As with the previous investigation, the overall likelihood of experiencing depressive symptoms in all the diabetes patients was higher for women than for men [12,26]. However, according to type of treatment, men had a greater risk of experiencing depressive symptoms than women [27].

In the male diabetes patients, in line with previous studies [15,22,23], showed that treatment with “oral agent and insulin injection” or “insulin injection” was more likely to cause depressive symptoms than treatment with “oral agent” alone. Consistently with a previous study, the patients who were using insulin showed more depressive symptoms. In the Sample description section of the study by Hermanns, of 376 diabetes patients, 53 had clinical depression. Of these patients, 16 were receiving type 1 diabetes treatment (30.2%); 11 were receiving type 2 diabetes treatment without insulin treatment (20.8%); and 26 were receiving type 2 diabetes treatment with insulin (49.0%). Of the 71 patients with subclinical depression, 31%, 22.5% and 46.5% were receiving the aforementioned treatments, respectively [23].

In female diabetes patients, oral agents and insulin injection significantly caused more depressive symptoms than oral agents alone according to statistics. Statistically significant results for insulin injection were difficult to obtain. However, the characteristics of the study population in our study showed that treatment with insulin injection resulted in a higher proportion of patients with depressive symptoms than treatment with oral agents. Previous studies showed that treatment with insulin injection affects depressive symptoms in diabetes patients. In a study that used PAID questionnaires to investigate the treatment methods for type 2 diabetes patients and their relationship to emotional distresses, the mean PAID score was 14.7 in the “diet alone” group and 17.8 in the “oral medication” group. “Insulin” had the highest score of 24.6 [15]. In Black’s study of older Mexican American diabetes patients, “insulin” had the highest OR value (OR = 1.83; 95% CI: 1.30–2.59), followed by “both insulin and OHA (oral hypoglycemic agent)” (OR = 1.79; 95% CI: 1.10–2.89). “OHAs” had the lowest OR value (OR = 0.72; 95% CI: 0.51–0.99) [22].

Our results for the male patients showed that the OR was highest for the oral agents and insulin injection group. That is, patients who took both oral agent medication and insulin injection tended to show depressive symptoms more often than patients who were treated only with insulin. In Welch’s study that used PAID, the mean PAID score was lowest for “diet treated” at 10.5 ± 12.8 and highest for “IDDM” at 32.9 ± 20.2. For the “NIDDM” groups, including “insulin treated” (26.4 ± 20.8) and “tablet treated” (24.9 ± 22.1), the score was 25.0 ± 20.9 [21]. Hermanns reported that PAID questionnaires are useful to examine the depression and emotional problems of diabetes patients [23]. Therefore, depressive symptoms in patients taking oral medications should not be overlooked. In conclusion, in this study, the diabetes patients who were undergoing diabetes treatment only showed differences in the degree of depressive symptoms depending on their sex and type of diabetes treatment, and all were vulnerable to the risk of depressive symptoms.

This study performed subgroup analyses of depressive symptoms and type of diabetes treatments according to age and body mass index. The results showed that the diabetes patient had a stronger association with depressive symptoms if they used both oral agents and insulin injection. Both male and female patients who received oral agents and insulin injection treatment were most likely to experience depressive symptoms at the age of 40–49 years. This is consistent with previous studies that showed that depression was more prevalent among diabetes patients aged 40–49 years [28]. Body mass index showed a different trend in the male and female patients. The normal-weight men had the lowest prevalence of depressive symptoms, while the women had the highest prevalence of depressive symptoms. Women were at higher and lower risks of experiencing depression when they were normal-weight and obese, respectively. This is considered to have been affected by the mental stress of the female to achieve normal weight due to the climate of Korean society, in which women care more about their weight than men.

Diabetes patients with depression have a lower quality of life and are less active in disease management than diabetes patients without depression [8,29]. Low quality of life and poor management of diseases worsens the depressive symptoms of diabetes patients with depression and eventually leads to a vicious circle that continues to degrade their health and quality of life. Therefore, managing depression in diabetics is very important. Our research is meaningful because we have again emphasized depression in diabetes patients by observing the association between the type of diabetes treatment and depressive symptoms among patients with diabetes. In addition, the bidirectional relationship between diabetes and depressive symptoms is well known [11]. However, regardless of whether depression adversely affects diabetes or diabetes adversely affects depression, all diabetes patients need treatments to manage their blood glucose. Therefore, our study is significant in that it demonstrates the association between the type of diabetes treatment and depressive symptoms. Furthermore, the strength of our study is that the depressive symptoms of diabetes patients were studied according to sex. Our research has the strength of its results being generalizable to the Korean adult population, as it used nationally representative data.

However, this study has some limitations, as it is conducted only with the aforementioned data. First, due to the bidirectional relationship between diabetes and depression [11], depression may occur before diabetes onset or treatment. On the contrary, diabetes can lead to or exacerbate depression, which can be caused by the burden of the disease (time, cost, effort) as well as by pathophysiological mechanisms [15,30]. Furthermore, if a patient with diabetes neglects to manage diabetes due to depression, the symptoms of diabetes may worsen. However, because the data is cross-sectional in design, the causal relationship between diabetes treatment and depressive symptoms remains unknown. In addition, due to the limitation of the survey items, it was not possible to know when diabetes and depression onset or when they were diagnosed and to know time, cost and efforts to manage diabetes. Therefore, it was not possible to detect patients who already had depression and to estimate the burden of disease in diabetes patients. Second, the presented data such as the depressive symptoms and health status variables were self-reported. Therefore, recall bias was possible. In addition, we used one question that asked about depressive symptoms rather than an objective depression inventory. Moreover, that question was about perceived depressive symptoms. Because there were no questions asked about the depression diagnosis or depression inventory, the responses to the feelings of sadness or depression for >2 weeks in a year were considered depressive symptoms. Third, the questions about diabetes were inadequate. None of the questions were about the type, duration, severity of diabetes, and glycemic control. Information about diabetes is important because it is closely related to the depressive symptoms and the strategy of treatment may vary accordingly. According to the Treatment Guideline for Diabetes in Korea, insulin injections are prescribed for type 1 diabetes, while oral hypoglycemic agents are prescribed first for type 2 diabetes, and if glycemic control is not good, use of both oral hypoglycemic agents and insulin injections is considered [31]. Therefore, type 2 diabetic patients who use both oral hypoglycemic agents and insulin injections may have more severe symptoms of diabetes. However, it was impossible to pinpoint the type and severity of diabetes in the data used in the study. In addition, diabetes duration is significantly associated with depression [32]. However, it could not be adjusted because it was also unknown. Moreover, our measures of the type of treatment for diabetes were based on single questions in the survey, which was too wide to know the details. Thus, the more detailed forms of insulin treatment (injection, pump [33]) or the ingredients and type of oral agents (metformin, thiazolidinediones, dipeptidyl peptidase-4, etc. [34]) could not be known. Also, how many times the patient took the oral agent or insulin injection could not be known. Finally, treatment efficacy according to disease management and medication adherence may be associated with depressive symptoms [35]. However, our study used cross-sectional data, and the questionnaire did not include the efficacy of the medicine, the severity of the disease, and the progress of the disease. Therefore, it is not possible to determine whether treatment efficacy influences the results. These limitations require further study. 

The bidirectional association of depressive symptoms with diabetes treatment effects and adherence is also well recognized [11,20]. To prevent diabetes complications, blood glucose control should be performed well, and to maintain blood glucose at a proper level, the risk of depression should be avoided. Accordingly, physicians must also manage diabetes patients’ mental health, including depressive symptoms. In addition, they should consider the depressive symptoms that diabetes patients may experience according to the type of diabetes treatment before the treatments are taken into account.

## 5. Conclusions

This study shows the difference in depressive symptoms according to the type of treatment among diabetes patients between the sexes. Depressive symptoms were more strongly associated with diabetes treatment in male patients than in female patients. Insulin injection was associated with depressive symptoms in diabetes patients. Oral agents and insulin injection had a significant correlation with depressive symptoms in diabetes patients. Oral agents and insulin injection were more likely to cause depressive symptoms than one form of treatment alone. Therefore, physicians should consider diabetes patients’ risk for developing depressive symptoms according to sex and the type of diabetes treatment. They should also include a process that provides mental support and treatment when managing all diabetes patients.

## Figures and Tables

**Table 1:** General characteristics of the study population with depressive symptoms.

Variables	Total		Male				*p*-Value *	Female				*p*-Value *
			Yes		No			Yes		No		
	*N*	%	*N*	%	*N*	%		*N*	%	*N*	%	
**Total**	99,752	100.0	2911	2.9	47,863	48.0		5207	5.2	43,771	43.9	
**Diabetes treatment**							<0.0001					<0.0001
Oral agents	90,182	90.4	2384	2.6	43,415	48.1		4471	5.0	39,912	44.3	
Insulin injection	2521	2.5	128	5.1	1273	50.5		157	6.2	963	38.2	
Oral agents and insulin injection	7049	7.1	399	5.7	3175	45.0		579	8.2	2896	41.1	
**Age**							<0.0001					0.0006
30–39	1396	1.4	79	5.7	780	55.9		67	4.8	470	33.7	
40–49	7528	7.5	286	3.8	4402	58.5		343	4.6	2497	33.2	
50–59	21,613	21.7	738	3.4	11,794	54.6		1011	4.7	8070	37.3	
60–69	32,185	32.3	850	2.6	15,683	48.7		1592	4.9	14,060	43.7	
70–79	29,785	29.9	793	2.7	12,594	42.3		1774	6.0	14,624	49.1	
80~	7245	7.3	165	2.3	2610	36.0		420	5.8	4050	55.9	
**Educational level**							<0.0001					0.2868
Elementary school or less	44,717	44.8	879	2.0	12,627	28.2		3337	7.5	27,874	62.3	
Middle school	18,166	18.2	607	3.3	9650	53.1		822	4.5	7087	39.0	
High school	24,168	24.2	925	3.8	15,642	64.7		831	3.4	6770	28.0	
College or over	12,701	12.7	500	3.9	9944	78.3		217	1.7	2040	16.1	
**Marital status**							<0.0001					<0.0001
Married-cohabit	72,774	73.0	2022	2.8	41,690	57.3		2768	3.8	26,294	36.1	
Married non-cohabitant or bereaved or divorced	25,153	25.2	716	2.8	4972	19.8		2371	9.4	17,094	68.0	
Unmarried	1825	1.8	173	9.5	1201	65.8		68	3.7	383	21.0	
**Occupation**							<0.0001					<0.0001
White collar	7244	7.3	246	3.4	5855	80.8		90	1.2	1053	14.5	
Pink collar	8424	8.4	164	1.9	3838	45.6		392	4.7	4030	47.8	
Blue collar	32,561	32.6	752	2.3	20,694	63.6		826	2.5	10,289	31.6	
Others	51,523	51.7	1749	3.4	17,476	33.9		3899	7.6	28,399	55.1	
**Monthly household income, 1000 KRW**							<0.0001					<0.0001
~999	35,161	35.2	1370	3.9	12,982	36.9		2683	7.6	18,126	51.6	
1000–1999	22,848	22.9	603	2.6	11,569	50.6		1092	4.8	9584	41.9	
2000–2999	15,729	15.8	377	2.4	8401	53.4		627	4.0	6324	40.2	
3000–3999	10,287	10.3	242	2.4	5714	55.5		321	3.1	4010	39.0	
4000–4999	6181	6.2	120	1.9	3535	57.2		212	3.4	2314	37.4	
5000~	9546	9.6	199	2.1	5662	59.3		272	2.8	3413	35.8	
**Residency region**							<0.0001					<0.0001
Urban area–large city	23,897	24.0	833	3.5	11,457	47.9		1458	6.1	10,149	42.5	
Urban area–small city	18,580	18.6	592	3.2	8790	47.3		1041	5.6	8157	43.9	
Rural area	57,275	57.4	1486	2.6	27,616	48.2		2708	4.7	25,465	44.5	
**Alcohol status**							0.5162					<0.0001
Ever	73,066	73.2	2640	3.6	43,232	59.2		3074	4.2	24,120	33.0	
Never	26,686	26.8	271	1.0	4631	17.4		2133	8.0	19,651	73.6	
**Smoking status**							0.0476					<0.0001
Ever	44,517	44.6	2413	5.4	38,972	87.5		623	1.4	2509	5.6	
Never	55,235	55.4	498	0.9	8891	16.1		4584	8.3	41,262	74.7	
**Walking activity**							<0.0001					<0.0001
Inactive	29,997	30.1	1062	3.5	14,062	46.9		1851	6.2	13,022	43.4	
Low	15,902	15.9	438	2.8	7364	46.3		873	5.5	7227	45.4	
Middle	13,996	14.0	372	2.7	6453	46.1		711	5.1	6460	46.2	
High	39,857	40.0	1039	2.6	19,984	50.1		1772	4.4	17,062	42.8	
**Perceived health status**							<0.0001					<0.0001
Good	13,026	13.1	160	1.2	8543	65.6		190	1.5	4133	31.7	
Normal	34,556	34.6	628	1.8	19,126	55.3		890	2.6	13,912	40.3	
Bad	52,170	52.3	2123	4.1	20,194	38.7		4127	7.9	25,726	49.3	
**Perceived stress level**							<0.0001					<0.0001
Very	25,887	26.0	1863	7.2	10,076	38.9		3443	13.3	10,505	40.6	
Less	45,860	46.0	832	1.8	22,838	49.8		1412	3.1	20,778	45.3	
Low	28,005	28.1	216	0.8	14,949	53.4		352	1.3	12,488	44.6	
**Body mass index (kg/m^2^)**							<0.0001					<0.0001
Underweight (~18.4)	2959	3.0	163	5.5	996	33.7		295	10.0	1505	50.9	
Normal weight (18.5–22.9)	33,982	34.1	1040	3.1	15,412	45.4		1794	5.3	15,736	46.3	
Overweight (23.0–24.9)	27,470	27.5	754	2.7	14,260	51.9		1198	4.4	11,258	41.0	
Obesity (25.0~)	35,341	35.4	954	2.7	17,195	48.7		1920	5.4	15,272	43.2	
**Hypertension diagnosis**							0.0027					0.0732
Yes	60,434	60.6	1752	2.9	27,451	45.4		3379	5.6	27,852	46.1	
No	39,318	39.4	1159	2.9	20,412	51.9		1828	4.6	15,919	40.5	
**Dyslipidemia diagnosis**							<0.0001					<0.0001
Yes	32,578	32.7	1078	3.3	13,732	42.2		2308	7.1	15,460	47.5	
No	67,174	67.3	1833	2.7	34,131	50.8		2899	4.3	28,311	42.1	
**Year**							<0.0001					<0.0001
2011	14,080	14.1	362	2.6	6660	47.3		627	4.5	6431	45.7	
2012	14,856	14.9	351	2.4	7209	48.5		663	4.5	6633	44.6	
2013	16,061	16.1	462	2.9	7753	48.3		812	5.1	7034	43.8	
2014	17,230	17.3	591	3.4	8204	47.6		1042	6.0	7393	42.9	
2015	18,280	18.3	582	3.2	8746	47.8		993	5.4	7959	43.5	
2016	19,245	19.3	563	2.9	9291	48.3		1070	5.6	8321	43.2	

* *p*-value is the result of the chi square test. Oral agents, Oral hypoglycemics; KRW, Korean Won;

**Table 2:** Factors associated with depressive symptoms in diabetes patients.

Variables	Male		Female	
	Adjusted OR	95% CI	Adjusted OR	95% CI
**Diabetes treatment**				
Oral agents	1.00	-	1.00	-
Insulin injection	1.27	(1.04–1.56)	1.17	(0.98–1.41)
Oral agents and insulin injection	1.41	(1.25–1.60)	1.35	(1.22–1.50)
**Age**				
30–39	1.00	-	1.00	-
40–49	0.71	(0.54–0.95)	1.20	(0.88–1.62)
50–59	0.73	(0.56–0.97)	1.17	(0.87–1.56)
60–69	0.63	(0.48–0.84)	0.92	(0.68–1.24)
70–79	0.58	(0.44–0.78)	0.82	(0.61–1.11)
80~	0.44	(0.32–0.62)	0.70	(0.51–0.96)
**Educational level**				
Elementary school or less	1.00	-	1.00	-
Middle school	1.01	(0.90–1.14)	1.05	(0.96–1.15)
High school	0.96	(0.86–1.08)	1.12	(1.01–1.24)
College or over	0.90	(0.79–1.04)	1.10	(0.92–1.31)
**Marital status**				
Married-cohabit	1.00	-	1.00	-
Married non-cohabitant or bereaved or divorced	1.93	(1.74–2.13)	1.40	(1.31–1.50)
Unmarried	1.41	(1.15–1.72)	1.26	(0.94–1.68)
**Occupation**				
White collar	1.00	-	1.00	-
Pink collar	0.92	(0.74–1.13)	1.14	(0.88–1.48)
Blue collar	0.86	(0.72–1.02)	1.12	(0.87–1.45)
Others	1.81	(1.52–2.16)	1.78	(1.39–2.27)
**Monthly household income, 1000 KRW**				
~999	1.00	-	1.00	-
1000–1999	0.69	(0.62–0.77)	0.82	(0.76–0.89)
2000–2999	0.63	(0.55–0.72)	0.73	(0.66–0.81)
3000–3999	0.58	(0.49–0.68)	0.58	(0.50–0.66)
4000–4999	0.48	(0.38–0.59)	0.71	(0.60–0.83)
5000~	0.49	(0.40–0.58)	0.61	(0.53–0.71)
**Residency region**				
Urban area–large city	1.00	-	1.00	-
Urban area–small city	0.84	(0.75–0.95)	0.84	(0.77–0.92)
Rural area	0.74	(0.67–0.81)	0.74	(0.69–0.80)
**Alcohol status**				
Ever	1.00	-	1.00	-
Never	0.90	(0.78–1.03)	0.89	(0.83–0.95)
**Smoking status**				
Ever	1.00	-	1.00	-
Never	1.10	(0.99–1.23)	0.67	(0.60–0.74)
**Walking activity**				
Inactive	1.00	-	1.00	-
Low	0.94	(0.83–1.07)	0.94	(0.86–1.03)
Middle	0.95	(0.83–1.09)	0.95	(0.86–1.05)
High	0.86	(0.78–0.95)	0.90	(0.83–0.97)
**Perceived health status**				
Good	1.00	-	1.00	-
Normal	1.44	(1.21–1.72)	1.14	(0.97–1.35)
Bad	2.53	(2.13–3.00)	1.90	(1.63–2.23)
**Perceived stress level**				
Very	1.00	-	1.00	-
Less	0.24	(0.22–0.26)	0.24	(0.22–0.25)
Low	0.09	(0.08–0.11)	0.10	(0.09–0.11)
**Body mass index (kg/m^2^)**				
Underweight (~18.4)	1.51	(1.24–1.84)	1.45	(1.25–1.68)
Normal weight (18.5–22.9)	1.00	-	1.00	-
Overweight (23.0–24.9)	0.92	(0.83–1.02)	0.96	(0.88–1.04)
Obesity (25.0~)	0.92	(0.83–1.02)	0.98	(0.91–1.06)
**Hypertension diagnosis**				
Yes	1.00	-	1.00	-
No	1.04	(0.96–1.14)	1.06	(0.99–1.13)
**Dyslipidemia diagnosis**				
Yes	1.00	-	1.00	-
No	0.75	(0.69–0.82)	0.78	(0.73–0.84)
**Year**				
2011	1.00	-	1.00	-
2012	0.89	(0.76–1.04)	1.06	(0.94–1.19)
2013	1.15	(0.99–1.34)	1.31	(1.16–1.47)
2014	1.36	(1.18–1.57)	1.58	(1.42–1.77)
2015	1.31	(1.13–1.51)	1.42	(1.27–1.59)
2016	1.17	(1.01–1.36)	1.52	(1.36–1.70)

Oral agents, Oral hypoglycemics; KRW, Korean Won.

**Table 3:** Subgroup analysis of depressive symptoms with the type of diabetes treatment by covariates *.

Variables	Diabetes Treatment				
	Oral Agents	Insulin Injection		Oral Agents and Insulin Injection	
	OR	OR	95% CI	OR	95% CI
**Male**					
**Age**					
30–39	+	+		+	
40–49	1.00	0.98	(0.51–1.89)	1.89	(1.28–2.81)
50–59	1.00	1.17	(0.78–1.74)	1.53	(1.19–1.96)
60–69	1.00	1.47	(1.02–2.10)	1.28	(1.02–1.62)
70–79	1.00	1.26	(0.84–1.91)	1.34	(1.05–1.70)
80~	-				
**Educational level**					
Elementary school or less	+	+		+	
Middle school	1.00	1.38	(0.90–2.12)	1.36	(1.03–1.78)
High school	1.00	1.53	(1.10–2.12)	1.53	(1.23–1.91)
College or over	1.00	0.95	(0.55–1.65)	1.75	(1.30–2.35)
**Walking activity**					
Inactive	1.00	1.31	(0.94–1.84)	1.55	(1.27–1.88)
Low	1.00	1.32	(0.76–2.32)	1.46	(1.03–2.06)
Middle	1.00	1.07	(0.60–1.89)	1.49	(1.04–2.13)
High	1.00	1.27	(0.91–1.78)	1.22	(0.98–1.52)
**Body mass index (kg/m^2^)**					
Underweight (~18.4)	1.00	2.13	(1.00–4.54)	1.66	(0.98–2.79)
Normal weight (18.5–22.9)	1.00	1.33	(0.98–1.81)	1.36	(1.11–1.67)
Overweight (23.0–24.9)	1.00	1.33	(0.88–2.02)	1.46	(1.13–1.89)
Obesity (25.0~)	1.00	0.96	(0.62–1.50)	1.41	(1.13–1.76)
**Female**					
**Age**					
30–39	+	+		+	
40–49	1.00	1.22	(0.67–2.22)	1.54	(1.04–2.28)
50–59	1.00	1.03	(0.67–1.57)	1.42	(1.12–1.80)
60–69	1.00	1.30	(0.93–1.83)	1.18	(0.98–1.42)
70–79	1.00	1.21	(0.86–1.71)	1.38	(1.15–1.64)
80~	-				
**Educational level**					
Elementary school or less	+	+		+	
Middle school	1.00	1.26	(0.80–1.98)	1.31	(1.01–1.70)
High school	1.00	1.09	(0.73–1.63)	1.45	(1.12–1.88)
College or over	1.00	0.76	(0.36–1.60)	1.84	(1.15–2.95)
**Walking activity**					
Inactive	1.00	1.32	(0.98–1.78)	1.45	(1.23–1.72)
Low	1.00	1.08	(0.66–1.76)	1.39	(1.08–1.79)
Middle	1.00	1.80	(1.11–2.89)	1.23	(0.91–1.66)
High	1.00	0.88	(0.62–1.24)	1.24	(1.04–1.49)
**Body mass index (kg/m^2^)**					
Underweight (~18.4)	1.00	1.14	(0.56–2.35)	1.27	(0.79–2.03)
Normal weight (18.5–22.9)	1.00	1.03	(0.76–1.40)	1.46	(1.22–1.74)
Overweight (23.0–24.9)	1.00	1.28	(0.87–1.88)	1.35	(1.08–1.67)
Obesity (25.0~)	1.00	1.30	(0.94–1.79)	1.27	(1.07–1.50)

+: The number is insufficient and cannot be calculated; * Age, sex, educational level, marital status, occupation, monthly household income, residency region, alcohol status, smoking status, perceived health status, walking activity, stress level, body mass index, hypertension diagnosis, dyslipidemia diagnosis, and year variables were adjusted. Oral agents, Oral hypoglycemics;
